# Differentiation of Minute Pulmonary Meningothelial-Like Nodules and Adenocarcinoma *In situ* with CT Radiomics

**DOI:** 10.2174/0115734056354822250217045544

**Published:** 2025-03-14

**Authors:** Yawen Zhang, Leilei Zhou, Jun Yao, Hai Xu, Yu-Chen Chen, Xiaomin Yong

**Affiliations:** 1 Department of Radiology, Nanjing Pukou People’s Hospital, Nanjing, China; 2 Department of Radiology, Nanjing First Hospital, Nanjing Medical University, Nanjing, China; 3 Department of Radiology, The First Affiliated Hospital of Nanjing Medical University, Nanjing, China

**Keywords:** Radiomics, minute pulmonary meningothelial-like nodules, adenocarcinoma *in situ*, computed tomography, Least absolute shrinkage and selection operator

## Abstract

**Background:**

An effective preoperative diagnosis between minute pulmonary meningothelial-like nodules (MPMNs) and adenocarcinoma *in situ* (AIS) can provide clinicians with appropriate treatment strategies.

**Objective:**

This study aimed to differentiate MPMNs from AIS *via* computed tomography (CT) radiomics approaches.

**Methods:**

Clinical and imaging data from fifty-one patients diagnosed with MPMNs and 55 patients diagnosed with AIS were collected from Jiangsu Province Hospital and Nanjing First Hospital from January 2016 to December 2022. All patients underwent chest CT scans before surgery. All CT images were segmented with ITK-SNAP software, and the radiomics features were further extracted with the Python PyRadiomics package. Least absolute shrinkage and selection operator (LASSO) regression analysis was used to select the optimal radiomics features for the construction of the model. The ROC curve was used to evaluate the diagnostic efficacy of the model.

**Results:**

After feature reduction and selection, 16 radiomics features were selected to construct the radiomics model. In the test set, the sensitivity, specificity, positive predictive value, negative predictive value, and accuracy of the k-nearest neighbor model were 87.5%, 88.9%, 96.9%, 77.8%, and 88.5%, respectively, and the AUC of the ROC curve was 0.969 (95% CI: 0.72-1.00).

**Conclusion:**

The CT radiomics model has exhibited high diagnostic value in the differential diagnosis between MPMNs and AIS.

## 1 INTRODUCTION

Minute pulmonary meningothelial-like nodules (MPMNs) were first reported by Korn *et al.* in 1960 [1]. Using electron microscopy and immunohistochemical staining techniques, Gaffey *et al.* [2] reported their cellular morphology, ultrastructure, and immunohistochemical staining to be remarkably similar to those of intracranial meningioma cells; hence, they were designated as MPMNs. They are typically incidentally discovered in routine pathological sections from surgical or autopsy specimens without clinical symptoms [1, 2]. To date, the histological origin and pathogenesis of MPMNs are still under exploration. Research has suggested that MPMNs originate from the meningeal epithelium and represent reactive changes in lung tissue [3, 4] rather than a true tumor requiring no specific treatment. On computed tomography (CT) images, MPMNs often appear as round or oval ground-glass opacity nodules with smooth borders, predominantly located beneath the pleura [5, 6].

Adenocarcinoma *in situ* (AIS) is an early stage of lung adenocarcinoma, and previous studies have indicated that sublobar resection can achieve or approach a five-year survival rate of nearly 100% [7]. An effective preoperative diagnosis between MPMNs and AIS can provide clinicians with appropriate treatment strategies, reducing unnecessary surgical trauma for some patients. Solitary or multiple MPMNs and AIS often present as ground-glass opacities on imaging, with no distinct morphological features that aid in identification, such as lobulation, speculation, or pleural involvement. Therefore, the diagnostic value of traditional diagnostic methods is limited. Previous studies on MPMNs have mostly been individual case reports or focused on pathological diagnosis and immunohistochemistry. Research in the field of radiology has primarily involved the analysis of morphological features of MPMNs on CT images, which are subject to significant subjective factors.

Currently, numerous studies have utilized radiomics techniques for disease diagnosis and prognosis prediction, including the prediction of pathological subtypes of ground-glass opacities [8-10], the differentiation of benign and malignant solitary pulmonary nodules [11, 12], and research on the invasiveness of pulmonary ground-glass opacities [13, 14]. Significant progress has been made in the discrimination, prognosis, and efficacy prediction of pulmonary nodules [15-17]. This study, for the first time, has made an attempt to utilize a radiomics approach by converting imaging data from MPMNs into finely quantified features, enabling further objective quantitative analysis of the lesions.

Therefore, this study speculated that CT radiomics model can effectively distinguish MPMNs from AIS, which can provide more accurate diagnostic and treatment plans for clinical practice.

## 2 MATERIALS AND METHODS

### 2.1 Clinical Data and Grouping

This study utilized datasets from two independent centers, First Affiliated Hospital of Nanjing Medical University and Nanjing First Hospital. A retrospective analysis of pulmonary pathological specimens collected from 98 patients with MPMNs from January 2016 to December 2022 was conducted. Combining preoperative chest CT images, 51 patients were further screened from the two hospitals, allowing for precise lesion localization on chest CT. Additionally, clinical data from 55 patients who were diagnosed with AIS and presented with ground-glass opacities were collected from Nanjing First Hospital. The selection of datasets from two independent centers made the research results more objective. Each patient’s detailed clinical history and relevant examination results are shown in Fig. (**1**) and Table **1**.

#### 2.1.1 Inclusion Criteria

The inclusion criteria comprised (1) all patients who have undergone preoperative CT examinations, and their imaging data were complete. (2) Thin-slice (≤ 1.50 mm) CT lung window images that can be used to accurately locate the lesion. (3) Confirmation through histopathological examination.

#### 2.1.2 Exclusion Criteria

The exclusion criteria were (1) patients whose clinical data or related examination results were incomplete, and (2) images with obvious artifacts.

### 2.2 Image Acquisition

Unenhanced chest CT was performed using two CT scanners: a 64-slice Siemens CT (Siemens Medical Solutions, Erlangen, Germany) or a 64-slice Philips CT (Ingenuity, Philips, Eindhoven, Netherlands). The patient was in the supine position with the arms raised to minimize scanning artifacts in the shoulders and upper limbs. The scanning range was from the thoracic entrance to 2 cm below the diaphragm. The CT scanning parameters were as follows: tube voltage, 120 KV; tube current, 100 mA; collimator width, 1.2mm; and pitch, 0.9. Lung window reconstructions with bone algorithms at layer thicknesses of 5 mm and 1.5 mm were carried out. Mediastinal window reconstructions were performed with a soft tissue algorithm at a layer thickness of 5 mm (lung window: window width 1200 HU, window level -600 HU; mediastinal window: window width 350 HU, window level 50 HU).

### 2.3 Lesion Segmentation

Fifty-one patients with MPMNs and 55 patients with AIS were enrolled in this study. Thin-slice images from the lung window of chest CT scans of all patients were imported into the software (ITK-SNAP) for image segmentation. A radiologist with over 5 years of diagnostic experience independently delineated the regions of interest (ROIs), making efforts to avoid areas with vascular bundles, calcifications, and other artifacts. Subsequently, a second radiologist with over 10 years of diagnostic experience checked the delineated images. After verification, the data were exported and saved in NII format.

### 2.4 Feature Extraction

After the completion of ROI delineation, the PyRadiomics toolkit was utilized to extract radiomics features, including first-order features, shape features, and texture features. Shape features were derived from the original images, while other features were obtained by applying various filters, such as wavelet, square, square root, gradient, logarithm, exponential, 2D local binary pattern (LBP-2D), and 3D local binary pattern (LBP-3D) filters. To extract texture features, wavelet filtering was applied to preprocessed CT images, involving transforming the volume of interest (VOI) into the wavelet domain, retaining low-pass (LLL) and high-pass (HHH) subbands, and assigning different weights to other subbands (LHL, LHH, LLH, HLL, HHL, and HLH).

### 2.5 Feature Selection

The high dimensionality of the data extracted from the regions of interest could lead to overfitting in the modeling process. Therefore, the least absolute shrinkage and selection operator (LASSO) was used to further reduce the dimension and adjust the weight λ. LASSO shrinks all regression coefficients to zero and sets the coefficients of irrelevant features to zero. Fivefold cross-validation was used, where the final value of λ yielded the minimum cross-validation error. The features with non-zero coefficient values were reserved for regression model fitting to construct the radiomics model.

### 2.6 Establishing Prediction Models

We utilized features extracted from lesion segmentation for data analysis and model construction, reflecting lesion information and predicting lesions. In the construction process, optimal radiomics features selected from the LASSO regression-based feature selection were employed. Eight machine learning algorithms were used to establish the models, including support vector machine, k-nearest neighbors, random forest, decision tree, XG boost, logistic regression, light GBM, and multilayer perceptron. Patients were randomly divided into training and testing groups in a 7:3 ratio. A 5-fold cross-validation set was used to evaluate and compare the performance of each model. The discriminatory ability of the model was assessed using metrics, such as the area under the receiver operating curve (ROC), accuracy, sensitivity, specificity, positive predictive value, and negative predictive value. An overview of the workflow of this study is shown in Fig. (**2**).

### 2.7 Statistical Analysis

Continuous variables are presented as mean ± standard deviation and they were analyzed using unpaired t-test. Categorical variables are expressed as absolute numbers (N) and relative frequencies (%) and were analyzed using the chi-square test. Differences at *p* < 0.05 were considered to indicate statistical significance.

## 3 RESULTS

### 3.1 Comparison of General Information

In total, 51 patients who were diagnosed with MPMNs were enrolled in this study, including 20 males and 31 females, with an average age of 58.71±9.26 years. Twenty-two patients had a history of smoking. Fifty-five patients diagnosed with AIS were enrolled, including 21 males and 34 females, with an average age of 54.68±13.28 years, and 23 patients had a history of smoking. There was no significant difference in age, sex, or smoking history between the two groups, as shown in Table **2**.

### 3.2 Selection of Radiomics Features

A total of 1837 radiomics features were extracted, and LASSO regression was employed to select 16 optimal radiomics features for model construction, as shown in Fig. (**3**). The weights of each feature coefficient are shown in Fig. (**4**). These features include 3 first-order histogram features and 13 texture features, comprising 1 gray level dependency matrix (GLDM), 1 gray level run length matrix (GLRLM), 4 gray level co-occurrence matrix (GLCM), and 7 gray level size zone matrix (GLSZM).

### 3.3 Model Construction and Diagnostic Performance of the Radiomics Model

Eight machine learning algorithms for constructing radiomics models were used in this study. Among them, random forest, extra trees, and XGBoost failed to construct effective models due to model complexity and overfitting. The KNN model and MLP model exhibited the highest sensitivity, specificity, accuracy, positive predictive value, and negative predictive value in the test set. Fig. (**5**) illustrates the comparison of ROC curves for different models. The KNN model demonstrated the highest AUC value in the test set. Therefore, the KNN model was selected as the optimal model. The performances of the different models on the test set were compared and are summarized in Table **2** and Fig. (**5**).

## 4 DISCUSSION

With the widespread application of high-resolution CT in health screening for the general population, the detection rate of ground-glass opacities in the lungs is increasing [18]. The traditional imaging diagnostic approach primarily involves morphological features, such as edges, shapes, vascular changes, air bronchogram signs, and pleural indentations, as well as quantitative features, such as lesion size, CT values, and volume. The morphological classification of lesions largely relies on the diagnostic skills of radiologists [9]. Quantitative features can be influenced by scanning parameters (such as measurement consistency), and studies have shown that ground-glass opacities smaller than 6 mm may overlap certain features [10]. Therefore, the accuracy of preoperative diagnosis is limited. Although dynamic monitoring of pulmonary nodules can allow timely intervention, it also poses the issue of excessive examinations [19]. Some nodules are distributed beneath the pleura, and the use of CT-guided percutaneous biopsy aids in making a definitive diagnosis. However, due to the small size of the nodules, the sensitivity of percutaneous biopsy is reduced, and there is a risk of complications associated with the biopsy procedure [20, 21]. In addition, due to tumor heterogeneity, it is not convincing to use only tiny tissues to represent the characteristics of the whole lesion [21, 22]. In this study, all 14 patients with MPMNs underwent surgical treatment due to misdiagnosis as having AIS, indicating the limited value of traditional imaging diagnosis. Therefore, there is a clinical need for a simple and reliable diagnostic tool to obtain additional information to assist in clinical diagnosis and treatment.

Radiomics involves the extraction and analysis of quantitative image features from medical images that are imperceptible to the naked eye of clinical practitioners [23-25]. It can capture numerous subtle and quantifiable features in medical images that are not visible to the naked eye, allowing for more sensitive detection of changes in the lesion area. Furthermore, models established using radiomics parameters are less influenced by the doctor's technical proficiency and subjective factors, providing a more objective basis for diagnostic conclusions. This offers a non-invasive and effective alternative method for disease diagnosis, differentiation, clinical treatment, and evaluation [26].

Due to their high contrast with surrounding lung tissues, lung nodules are considered more suitable for radiomics studies because of their high segmentation accuracy [27]. Radiomics is now widely applied in the detection and staging of lung cancer, as well as in predicting patient responses to treatment. Zhao *et al.* [9] used a random forest model to predict the pathological subtypes of lung adenocarcinoma presenting as ground-glass opacities. The results showed that the radiomic prediction model, which combines random forest with hyperparameter tuning, could effectively differentiate between invasive adenocarcinoma (IAC) and minimally invasive adenocar-cinoma (MIA), demonstrating clinical applicability. Liu *et al.* [28] proposed the use of radiomics features to predict the malignancy of lung nodules, achieving an accuracy of 81%, sensitivity of 76.2%, and specificity of 91.7%, indicating good discriminative capabilities of these features. Aerts *et al.* [29] reported that radiomics features assessed before treatment were helpful in predicting the response of non-small cell lung cancer (NSCLC) patients to EGFR mutations. Bogowicz *et al.* [30] noted CT radiomics to be a promising biomarker for predicting outcomes both before and during treatment.

In this study, a total of 16 radiomics features were ultimately extracted, including 3 first-order histogram features and 13 texture features. These features lack morphological characteristics, and the analysis suggested that this may be due to the small volume of the nodules, all of which presented as ground-glass density shadows and lacked typical morphological features. First-order histogram features, including median features, minimum feature values, and root mean squares, are the simplest statistical descriptors based on global grayscale data. These three features describe the 3D size of the lesions. Texture features represent the difference in ROI grayscale, and grayscale variations in the image are an important manifestation of lesion heterogeneity [24]. Using the selected radiomics features to construct a model, the sensitivity, specificity, and accuracy in the testing groups were relatively high. This indicated that the model could effectively discriminate between MPMNs and AIS, with a high positive rate and low likelihood of missing cases relative to traditional imaging diagnostic methods, allowing clinicians to provide more accurate treatment methods and avoid unnecessary surgery for patients.

## 5 LIMITATIONS

There were several limitations in this study. First, the sample size was relatively small. Second, this study was a retrospective study, and selection bias may have been inevitable in sample selection. Additionally, as the lesions are relatively small in volume, when determining the specific location through CT images, even with preoperative CT-guided localization images, there may be deviations. However, this study lays the foundation for future research. Future studies can expand the sample size and further increase the validation set to confirm the generalizability and robustness of the results.

## CONCLUSION

The model constructed based on CT radiomics could effectively discriminate between MPMNs and AIS patients, providing a basis for the selection of clinical diagnostic and treatment approaches.

## AUTHORS’ CONTRIBUTIONS

Y.Z. and L.Z. conceptualized the study and wrote the original draft; L.Z. contributed to methodology, software curation, resource allocation, data curation, and visualization; J.Y. validated the findings; H.X. performed formal analysis and investigation; Y.C. and X.Y. contributed to writing, review, and editing, and supervision; Y.C. contributed to project administration and funding acquisition. All authors have read and agreed to the published version of the manuscript.

## Figures and Tables

**Fig. (1):**
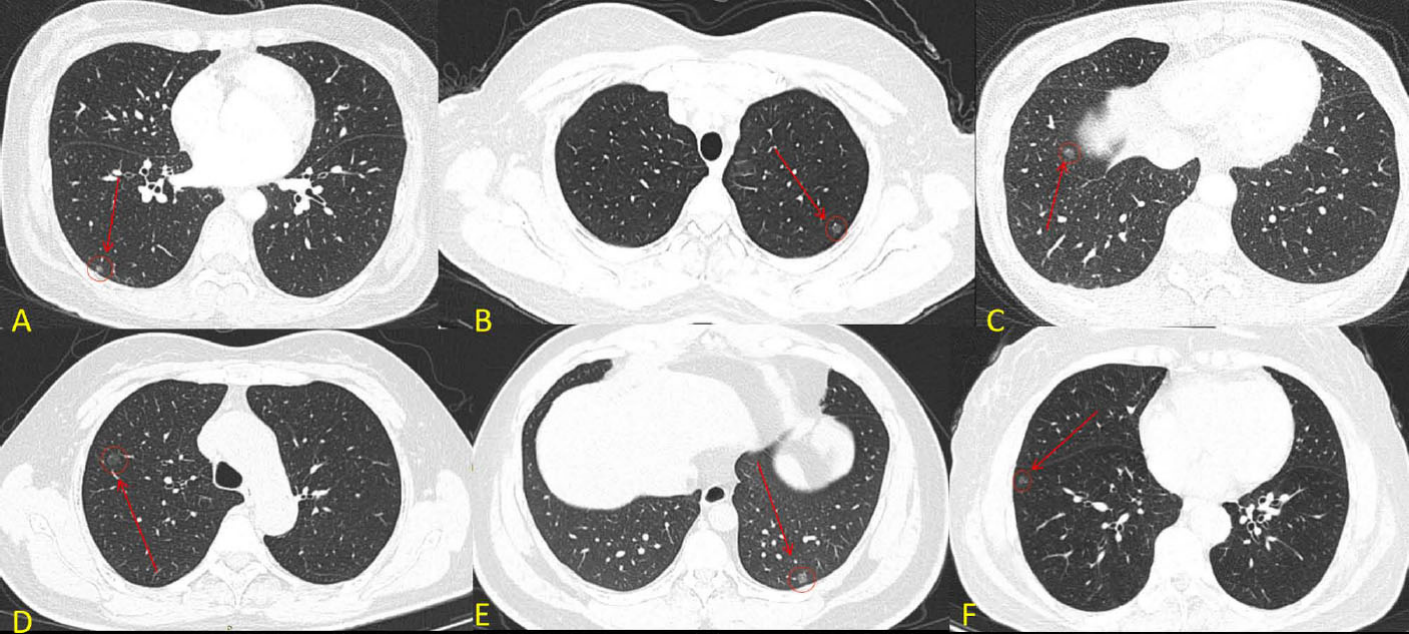
CT images of MPMNs and AIS. **A**): Female, 48 years old, MPMNs in the right lower lung; **B**): Female, 53 years old, MPMNs in the left lower lung; **C**): Female, 48 years old, MPMNs in the right lower lung. **D**): Female, 61 years old, AIS in the right lower lung; **E**): Male, 31 years old, AIS in the left lower lung; **F**): Female, 61 years old, AIS in the right lower lung.

**Fig. (2):**
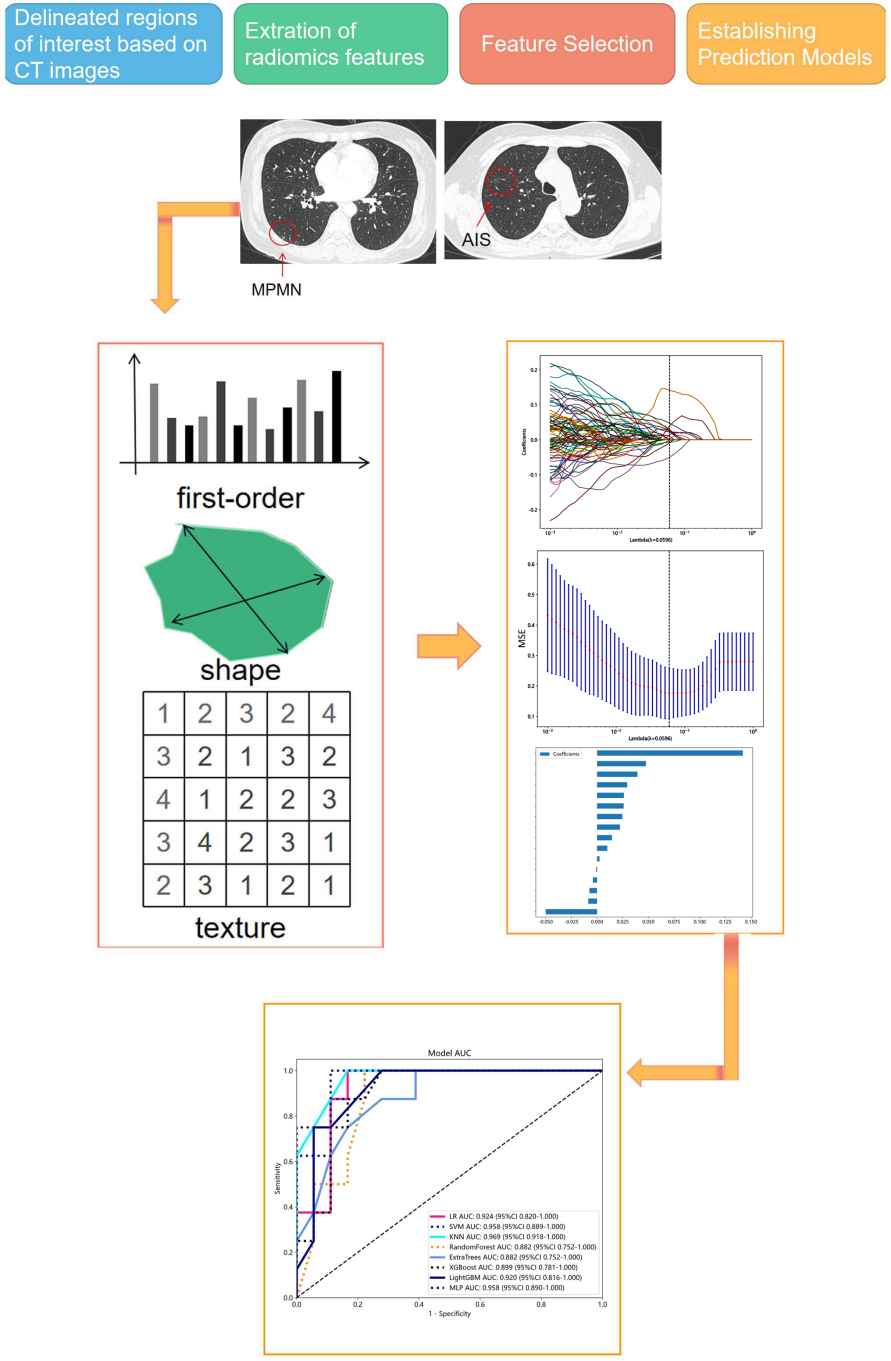
The workflow of this study. Radiologists manually delineated the region of interest (ROI) of pulmonary nodules, extracted radiomics features for model construction, and used the area under the curve (AUC) to evaluate the diagnostic value of the model.

**Fig. (3):**
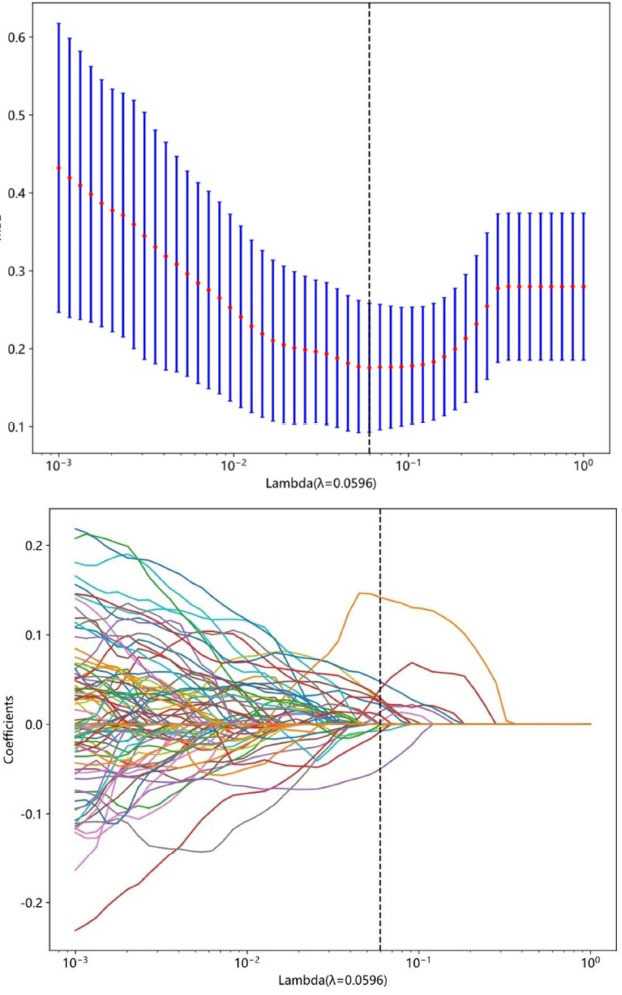
LASSO regression analysis.

**Fig. (4):**
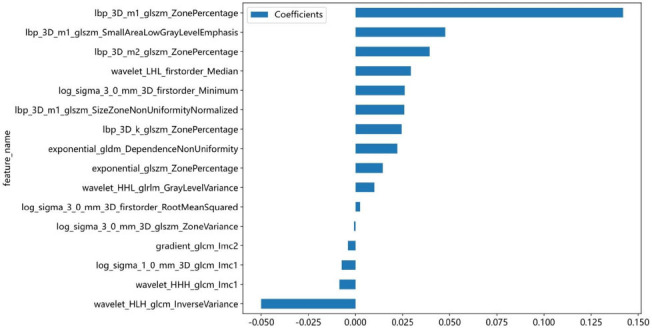
The proportions of optimal radiomics features.

**Fig. (5):**
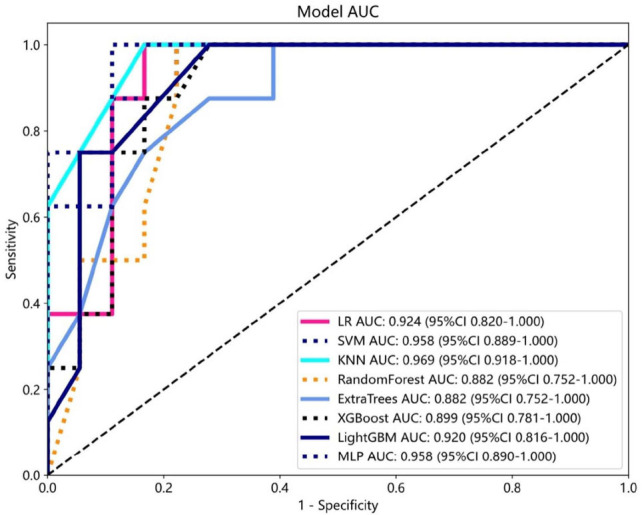
The comparison of ROC curves for different models in the test set.

**Table 1:** Comparison of general information between MPMNs and AIS.

**Clinical Information**	**MPMNs (n=51) **	**AIS (n=55) **	***P*-value**
Age (year)	58.71±9.26	57.68±13.28	0.883
Sex (male/female)	20/31	21/34	0.913
Smoking (n %)	22 (43.1%)	23 (41.8%)	0.891

**Table 2:** Predictive performance of the five machine learning algorithms on test set.

**Models **	**Sensitivity **	**Specificity **	**Accuracy **	**AUC **	**PPV **	**NPV**
LR	0.875	0.833	0.846	0.924	0.7	0.937
SVM	0.875	0.778	0.808	0.958	0.636	0.933
KNN	0.875	0.889	0.885	0.969	0.778	0.941
Light GNM	0.75	0.889	0.846	0.92	0.75	0.889
MLP	0.875	0.889	0.885	0.958	0.778	0.941

**Abbreviations:** AUC, area under the curve; PPV, positive predictive value; NPV, negative predictive value; LR, logistic regression; SVM, support vector machine; KNN, k-nearest neighbor; LightGBM, light gradient boosting machine; MLP, multilayer perceptron.

## Data Availability

The data supporting the results of this study will be available from the corresponding authors [Y.C.C.] and [X.Y.] upon reasonable request.

## LIST OF ABBREVIATIONS

ROC: Receiver Operating Curve
LASSO: Least Absolute Shrinkage and Selection Operator
CT: Computed Tomography
MPMNs: Minute Pulmonary Meningothelial-like Nodules
AIS: Adenocarcinoma *in situ*
VOI: Volume of interet
IAC: Invasive Adenocarcinoma

## STANDARDS OF REPORTING

STROBE guidelines were followed.
